# Editorial for the Special Issue: Diagnosis, Epidemiology and Transmission Dynamics of *Cryptosporidium* spp. and *Giardia duodenalis*

**DOI:** 10.3390/pathogens11020141

**Published:** 2022-01-24

**Authors:** Pamela C. Köster, David González-Barrio, David Carmena

**Affiliations:** Parasitology Reference and Research Laboratory, Spanish National Centre for Microbiology, Majadahonda, 28220 Madrid, Spain; pamelakster@yahoo.com (P.C.K.); dacarmena@isciii.es (D.C.)

*Cryptosporidium* spp. and *Giardia duodenalis* are major contributors to the global burden of diarrhoeal disease, primarily affecting young children in poor-resource settings. Cryptosporidiosis ranks second to rotavirus infection as a cause of life-threatening diarrhoea in children younger than two years of age, in sub-Saharan Africa and south Asia [1]. In contrast, giardiasis is rarely a cause of childhood mortality, but the disease has been consistently associated with growth faltering and cognitive impairment in malnourished children [2]. Intriguingly, large epidemiological studies, based on case-control data, have evidenced that *G. duodenalis* was more frequent among controls (individuals without diarrhoea) than in cases (individuals with diarrhoea) [1,3,4]. This finding has been interpreted by some authors as evidence in favour of a potential “protective” effect of the parasite against diarrhoea, and explains why *G. duodenalis* infections are systematically absent in global burden estimations of diarrhoeal diseases [5].

At least 44 *Cryptosporidium* species and more than 120 genotypes, as well as nine *Giardia* species are currently recognised [6]. Only three *Cryptosporidium* species (mainly anthroponotic *C. hominis* and zoonotic *C. parvum* and *C. meleagridis*) are responsible for most (circa 95%) human cases of cryptosporidiosis reported globally. *Giardia duodenalis* is the only *Giardia* species infective to humans, comprising eight (A–H) distinct genetic groups (the so-called assemblages) of which zoonotic assemblages A and B are commonly reported to infect humans [6]. Because of the large number of morphologically identical (but genetically different) species/genotypes, within both groups of protozoa, molecular-based tools, including Sanger sequencing, are needed for detection, differentiation, and subtyping purposes [7]. Molecular data are also essential to characterize the transmission dynamics of *Cryptosporidium* spp. and *G. duodenalis*, including for the identification of sources of infection and spread pathways, the tracking of virulent genetic variants or the assessment of zoonotic potential. The most common genetic markers used in subtyping analyses include the *Cryptosporidium* 60-kDa glycoprotein (*gp60*) gene, and the *G. duodenalis* β-giardin (*bg*), glutamate dehydrogenase (*gdh*), and triosephosphate isomerase (*tpi*) genes [7].

This special issue includes 14 papers that made important contributions in expanding our current knowledge, on aspects relevant to the diagnosis and epidemiology of *Cryptosporidium* spp. and *G. duodenalis*. These include the usefulness of PCR-based methods, for the first-line routine diagnosis of *Cryptosporidium* spp. in clinical settings [8], or for enlarging the available arsenal of subtyping tools, to assess *Cryptosporidium* intra-species genetic diversity in canine-adapted *C. felis* [9] and ovine-adapted *C. xiaoi* [10]. We were lucky enough to receive relevant contributions dealing with the human molecular epidemiology of *Cryptosporidium* spp. and *G. duodenalis* in a low-income country, such as Mozambique [11,12,13], in medium-income countries, such as Brazil and China [14,15], and in a high-income country, such as Sweden [16]. Finally, this special issue also included molecular epidemiological studies directed to assess the occurrence, genetic diversity and zoonotic potential of *Cryptosporidium* spp. and *G. duodenalis* in pet dogs and cats [17], livestock [18], and farmed rabbits and rats [19,20,21], mostly in China.

In their study, Costa et al. [8] evaluated the diagnostic performance of four “in-house” and four commercial PCR methods for the detection of *Cryptosporidium* spp. against a panel of selected stool samples, belonging to the collection of a French reference laboratory. The authors identified significant differences in the performance of the compared assays, highlighting the need for proper validation and standardization before routine clinical use. An asset of this study was the inclusion of less frequent or rare *Cryptosporidium* species and genotypes (*C. cuniculus*, *C. felis*, *C. meleagridis*, *C. ubiquitum*, *C.* sp. chipmunk genotype), in addition to *C. hominis* and *C. parvum*. All four commercial assays were able to identify all *Cryptosporidium* species and genotypes evaluated, but this was not always the case for the “in-house” protocols.

Li et al. [9] confirmed and expanded the usefulness of the 60-kDa glycoprotein (*gp60*) gene subtyping tool for assessing the genetic diversity within *C. felis*, initially developed by Rojas-Lopez et al. [22]. To do so, the authors identified the subtypes of 20 *C. felis* isolates, obtained from stray, sheltered, and pet cats, in Guandong province and Shanghai city in China. A high intra-species genetic diversity was observed, resulting in the identification of 13 novel and two known subtypes of the parasite. The main contribution of this survey was the demonstration that many of these genetic variants were shared between cats and humans, strongly suggesting that there could be cross-species transmission of *C. felis*. Furthermore, Fan et al. [10] developed a new *gp60* subtyping tool for *C. xiaoi*, a *Cryptosporidium* species adapted to infect sheep and goats, for which this tool was previously lacking. The authors tested 355 *C. xiaoi*-positive samples from Chinese sheep and goats and found an extremely large intra-species genetic diversity, resulting in the identification of 12 (XXIIIa to XXIIIl) subtype families. This study complements and expands the available *gp60* subtyping toolbox, including protocols adapted to *Cryptosporidium* species, such as *C. hominis* and *C. parvum* [23], *C. fayeri* [24], *C. meleagridis* [25], *C. ubiquitum* [26], *C. viatorum* [27], *C. felis* [22], *C. ryanae* [28], *C. canis* [29], and *C. bovis* [30].

This special issue brings together new epidemiological data that contribute to improving our understanding on the current situation of human cryptosporidiosis in Mozambique, one of the least developed countries in sub-Saharan Africa. In a seminal molecular-based study conducted in Zambézia province, Muadica et al. [11] investigated the presence and genetic diversity of *Cryptosporidium* spp., and other intestinal micro-eukaryotes, in a large population (*n =* 1093) of asymptomatic and symptomatic schoolchildren. The authors found a low prevalence of *Cryptosporidium* infections (1.6%) and managed to genotype 13 isolates of the parasite. Three species (*C. hominis*, *C. parvum*, and *C. felis*) were identified at equal rates (31% each), with *C. viatorum* being detected in 7% of cases. These preliminary results were further confirmed and expanded in a subsequent retrospective study, conducted by Messa et al. [12], taking advantage of a large panel of *Cryptosporidium*-confirmed DNA samples (*n* = 190), obtained during the Global Enteric Multicenter Study (GEMS) at the Manhiça district in the Maputo province [1]. The GEMS project was specifically designed as a case-control study to determine the etiology and population-based burden of paediatric diarrheal disease in sub-Saharan Africa and South Asia [31]. In their study, Messa et al. [12] identified three *Cryptosporidium* species including *C. hominis* (73%), *C. parvum* (23%), and *C. meleagridis* (4%). Both *C. hominis* and *C. parvum* were more prevalent among children with diarrhoea than in children without it (48% vs. 33%). A large intra-species genetic variability was observed within *C. hominis* (*gp60* subtype families Ia, Ib, Id, Ie, and If) and *C. parvum* (*gp60* subtype families IIb, IIc, IIe, and IIi) but not within *C. meleagridis* (*gp60* subtype family IIIb). Molecular genotyping data, provided by Cossa-Moiane et al. [13], in young children (*n* = 319) presenting with diarrhoea at hospital settings in the Maputo province, pointed in the same direction. In this study, a microscopy-based *Cryptosporidium* prevalence of 11% was obtained. In addition, typing results were available from a subset (*n* = 29) of these *Cryptosporidium*-positive samples, confirming the predominance of *C. hominis* (93%) over *C. parvum* (3%). A mixed infection of *C. hominis* and *C. parvum* was also detected (3%). Taken together, these three studies strongly suggest that human cryptosporidiosis in Mozambique is mainly of anthropic nature, although domestic dogs, cattle, and avian species can act as source of human infection in certain areas. This situation reflects the coexistence of different transmission pathways of cryptosporidiosis in the country.

Understanding the public health significance of emerging diarrhoea-causing microeukaryotes, including *Cryptosporidium* spp. and *G. duodenalis*, is increasingly attracting research interest in rapidly developing countries, such as Brazil and China [32,33]. In the former country, Köster et al. [14] investigated the occurrence and genetic diversity of *G. duodenalis* in a community survey of indigenous people (*n* = 574) from the Brazilian Amazon. During the four consecutive sampling campaigns of the study, *G. duodenalis* prevalence rates varied from 13–22% and primarily affected individuals younger than 15 years of age. Near 75% of the infections were attributed to the assemblage B of the parasite. Remarkably, no association between the *G. duodenalis* genotype and the occurrence of diarrhoea could be demonstrated. This finding is in agreement with the results obtained in a recent study conducted in Mozambican children younger than five years of age [34]. Considered together, these findings indicate that the parasite genotype does not suffice to explain, per se, the progression from infection to disease. Little information on the molecular variability of *Cryptosporidium* spp. and *G. duodenalis* is still available from Chinese human populations. Zhang et al. [15] attempted to overcome this gap in knowledge by analysing stool samples (*n* = 507) from randomly selected individuals, with and without gastrointestinal manifestations, seeking medical attention at hospital settings in the Yunnan Province. Interestingly, no *Cryptosporidium* infections were identified in the surveyed clinical population, whereas a low *G. duodenalis* occurrence rate (2%) was found. Sequence analyses of the *G. duodenalis*-positive isolates confirmed that assemblage A was far more prevalent than assemblage B (90% vs. 10%, respectively). The large, geographical-dependent difference in assemblage frequencies, documented in the studies mentioned above, may be indicative of different sources of infection and transmission routes.

*Cryptosporidium* spp. and *G. duodenalis* are a public health concern, not only in low- and medium-income countries, but also in developed nations [35]. In their study, Lebbad et al. [16] retrospectively genotyped *Cryptosporidium*-positive stool samples (*n* = 398) collected in 12 of the 21 regional laboratories that carry out routine parasitological diagnosis in Sweden. The cohort of stool samples analysed included patients that acquired the infection in the country and abroad. The authors identified 12 distinct *Cryptosporidium* species/genotypes, with *C. parvum* (75%) and *C. hominis* (12%) accounting for the majority of the cases identified. A very large intra-species genetic diversity was detected, allowing the identification of 29 *gp60* subtype families including four novel ones (*C. parvum* IIr, IIs, IIt, and *Cryptosporidium* horse genotype VIc). The authors also reported a human infection by rodent-adapted *C. ditrichi*, a *Cryptosporidium* species very rarely found in humans [36]. Another major contribution to this survey was the demonstration that almost 8% of human cryptosporidiosis cases in Sweden had a zoonotic origin. However, it is still unclear whether some of these findings (e.g., *C. erinacei*, *C. ditrichi*, *C.* horse genotype) correspond to true or spurious infections.

From the human molecular data presented above, it is clear now that zoonotic transmission is a significant route of *Cryptosporidium* spp. and *G. duodenalis*, spreading in several settings, either in developing or developed nations. Human infections can arise through direct contact with infected animals, or through accidental ingestion of contaminated water or food with the faecal material of these animals [37,38,39]. This special issue included five articles dealing with the potential role of companion, livestock, and farmed animals as potential sources of human cryptosporidiosis and giardiasis. Most of these studies were conducted in China. In the first one, Wang et al. [17] investigated the presence and genetic diversity of *Cryptosporidium* spp. and *G. duodenalis* in faecal samples from pet dogs (*n* = 262) and cats (*n* = 171), collected in veterinary clinics, markets and shelters, from the Yunnan province. Reported infection rates for *G. duodenalis* and *Cryptosporidium* spp. were 14% and 5% in dogs, and 1% each in cats, respectively. Sequence analyses revealed that dogs were infected only by canine-adapted *Cryptosporidium* (*C. canis*) and *G. duodenalis* (assemblages C and D) species/genotypes. Similarly, cats were infected by feline-adapted *C. felis* and *G. duodenalis* assemblage F. These data indicate that pet dogs and cats play a marginal role as sources of human cryptosporidiosis and giardiasis. It should be noted that most molecular-based surveys conducted so far, failed to demonstrate zoonotic transmission events between pet dogs/cats and humans [40,41]. In another survey, Cao et al. [18] assessed the occurrence and molecular diversity of *Cryptosporidium* spp. in faecal samples (*n* = 476) from Bactrian camels in the Xinjiang Uygur Autonomous Region. In this Chinese area, the Bactrian camel is one of the few large animal species suitable for livestock production, providing clothing, milk, meat, and transport for many people. The authors found a PCR-based prevalence of 8% and identified six different *Cryptosporidium* species circulating in the surveyed camel population. Of them, *C. andersoni* (67%) and *C. parvum* (17%) accounted for eight out of ten infections. Other, less frequent species, included *C. occultus*, *C. ubiquitum*, *C. hominis*, and *C. bovis*. The study represents the first report of *C. hominis* (*gp60* subtype family Ik) in Bactrian camels, expanding the known range of suitable hosts for this pathogen in China that already included cattle [42], donkeys [43], horses [43], and non-human primates [44], beside humans. Some authors have interpreted all this information as evidence in support of considering *C. hominis* as a zoonotic species [45]. Regarding intensive animal farming, Cui et al. [19] investigated the presence, molecular variability and zoonotic potential of *G. duodenalis* in coypus (*n* =308), reared in fur farms in six Chinese provinces/autonomous regions. The parasite was detected in all farms investigated at variable infection rates, ranging from 1–29%. Subtyping data was available for 38 isolates, of which 95% were assigned to assemblage B and the remaining 5% to assemblage A. These data indicate that giardiasis could be an occupational risk for those individuals working in close contact with the farmed coypus or their excreta. In a similar study conducted in farmed bamboo rats (*n* = 724), in Guangdong province, Li et al. [20] identified *Cryptosporidium* infections in 12% of the investigated animals. Sequence analyses of the isolates positive to the parasite allowed for the identification of five distinct *Cryptosporidium* species/genotypes, with *C.* bamboo rat genotype I (56%) and *C. parvum* (35%) being the most prevalent genetic variants found. The remaining positive samples belonged to *C.* bamboo rat genotype III (6%), *C. occultus* (2), and *C. muris* (1%). All *C. parvum* isolates were assigned to the rare *gp60* subtype families IIo and IIp (not previously identified in human cryptosporidiosis cases). Taken together, these data indicate that farmed bamboo rats were infected by rodent-adapted *Cryptosporidium* species with little, or no zoonotic potential. Finally, Naguib et al. [21] provided novel data on the presence and genetic variability of *Cryptosporidium* spp. in farmed rabbits in Egypt, a country where the molecular epidemiology of this protozoan parasite is poorly understood. The authors collected and analysed faecal samples (*n* = 235) from nine rabbit farms, located in three Egyptian provinces. *Cryptosporidium* infections were confirmed in eight out of the nine farms sampled at variable rates, ranging from 4% to 24%. All *Cryptosporidium*-positive isolates generated (*n* = 28) were identified as *C. cuniculus* and belonged to the *gp60* subtype family Vb. Although *C. cuniculus* is known to be particularly adapted to infect domestic and wild leporids, it has also been identified in clinical human cases [46] and in waterborne outbreaks of cryptosporidiosis [47] and is regarded as an emerging pathogen to humans.
